# Metabolomics Biomarkers for Detection of Colorectal Neoplasms: A Systematic Review

**DOI:** 10.3390/cancers10080246

**Published:** 2018-07-27

**Authors:** Vanessa Erben, Megha Bhardwaj, Petra Schrotz-King, Hermann Brenner

**Affiliations:** 1 Division of Preventive Oncology, German Cancer Research Center (DKFZ) and National Center for Tumor Diseases (NCT), 69120 Heidelberg, Germany; vanessa.erben@nct-heidelberg.de (V.E.); megha.bhardwaj@nct-heidelberg.de (M.B.); petra.schrotz-king@nct-heidelberg.de (P.S.-K.); 2 Medical Faculty Heidelberg, Heidelberg University, 69120 Heidelberg, Germany; 3 Division of Clinical Epidemiology and Aging Research, German Cancer Research Center (DKFZ), 69120 Heidelberg, Germany; 4 German Cancer Consortium (DKTK), 69120 Heidelberg, Germany

**Keywords:** metabolomics, biomarkers, early detection, colorectal neoplasms, sensitivity, specificity, human bio-fluids

## Abstract

Background: Several approaches have been suggested to be useful in the early detection of colorectal neoplasms. Since metabolites are closely related to the phenotype and are available from different human bio-fluids, metabolomics are candidates for non-invasive early detection of colorectal neoplasms. Objectives: We aimed to summarize current knowledge on performance characteristics of metabolomics biomarkers that are potentially applicable in a screening setting for the early detection of colorectal neoplasms. Design: We conducted a systematic literature search in PubMed and Web of Science and searched for biomarkers for the early detection of colorectal neoplasms in easy-to-collect human bio-fluids. Information on study design and performance characteristics for diagnostic accuracy was extracted. Results: Finally, we included 41 studies in our analysis investigating biomarkers in different bio-fluids (blood, urine, and feces). Although single metabolites mostly had limited ability to distinguish people with and without colorectal neoplasms, promising results were reported for metabolite panels, especially amino acid panels in blood samples, as well as nucleosides in urine samples in several studies. However, validation of the results is limited. Conclusions: Panels of metabolites consisting of amino acids in blood and nucleosides in urinary samples might be useful biomarkers for early detection of advanced colorectal neoplasms. However, to make metabolomic biomarkers clinically applicable, future research in larger studies and external validation of the results is required.

## 1. Introduction

Colorectal cancer (CRC) is the third most common cancer worldwide among men and the second most common in females [1]. Although it progresses slowly over a long period of time, it is often detected at advanced stages when prognosis is already poor [2]. CRC often develops without obvious early symptoms, and a large proportion of the at-risk population does not take advantage of screening offers. Colonoscopy—today’s gold standard for the early detection and removal of precancerous lesions—is invasive, inconvenient for the patients, and costly [3]. Established non-invasive tests, such as fecal occult blood tests (FOBT), have high specificity but limited sensitivity, especially with respect to the detection of precursors of CRC, such as adenomas. 

Therefore, there is need for the discovery of novel non-invasive screening methods and biomarkers that can identify CRC and its precursors in easily accessible biospecimens [4]. Recently, early detection of CRC in blood samples has drawn increasing attention among researchers. For example, the US Food and Drug Administration (FDA) recently approved a test that investigates methylation patterns in free circulating DNA in plasma [5]. One promising approach for biomarker detection with high diagnostic performance is metabolomics, the analysis of small molecular weight metabolites of different biochemical classes in the body [6]. Metabolites are closely related to the phenotype and mirrors the processes that are happening in the cell or the organism. The most readily accessible bio-samples such as stool, urine, and blood have great potential for discovery of early cancer biomarkers or even precursors such as adenomas [6]. On the other hand, the metabolomic profile is highly independent from influencing factors such as the environment or diet, which makes the application in biomarker discovery challenging [7].

A number of studies have assessed the potential of metabolomics for the early detection of adenomas and CRC and partly reported very promising results [8,9,10,11]. However, the large heterogeneity in study populations, biospecimen, analysis, analytical and statistical methods, and the extent of internal and external validation make comprehensive evaluation of the current state of knowledge difficult. We therefore carried out a systematic review in order to provide a comprehensive overview on the current state of knowledge in this promising field.

## 2. Methods

### 2.1. Systematic Literature Research

We conducted systematic literature research on biomarkers in non-invasive (urine, stool) or minimally invasive (blood) collectable bio-samples that might be promising for early detection of colorectal neoplasms. The search was conducted in PubMed and Web of Science on 26 April 2018 with the following search terms ((biomarker OR biomarkers OR metabolite OR metabolites OR metabolome OR metabolomic OR metabolomics OR metabolic) AND (Urine OR urinary OR blood OR plasma OR serum OR sera OR stool OR fecal OR feces OR urine-based OR blood-based OR plasma-based OR serum-based) AND (sensitivity OR specificity OR accuracy OR auc OR roc OR performance OR detection OR predictivity OR receiver operating characteristic) AND (“Colorectal neoplasm” OR “colon neoplasm” OR “colonic neoplasm” OR “Rectal Neoplasm” OR “colorectal cancer” OR “colon cancer” OR “colonic cancer” OR CRC OR “Colorectal tumor” OR “colon tumor” OR “colonic tumor” OR adenoma)) searching for “title/abstract” in the PubMed database specifically. We used the Preferred Reporting Items for Systematic Reviews and Meta-Analyses (PRISMA) statement flow diagram for systematic reviews to show at each phase the number of records and reasons for exclusion [12]. Cross references identified from original papers and reviews were also included. 

### 2.2. Exclusion Criteria

After the removal of study duplicates and articles that were not available in English language, we screened remaining titles and abstracts for eligible studies according to the predefined criteria. We removed records when the topics were not related to the review question (e.g., when the articles addressed other cancer types or other diseases). Furthermore, we excluded treatment trials and articles that used approaches other than metabolomics or focused on advanced or metastatic CRC cases. We looked at the remaining studies in more detail and further excluded reviews and papers not related to the topic (e.g., investigation on fecal immunochemical tests, volatile compounds) or studies using tissue samples rather than blood, urine, or stool samples for biomarker detection. Studies that did not contain enough statistical data or did not report on diagnostic performance were also not eligible. 

### 2.3. Data Extraction

We extracted details on study design and characteristics (year, type of study participants, samples size, gender distribution, and stage distribution) and on the metabolomics pattern found in the different bio-fluids, as well as the corresponding diagnostic performance characteristics (sensitivity, specificity, area under the curve (AUC), and *p*-value) from each article. If sensitivity and specificity were not reported directly, we used additional information to calculate these values whenever possible. Data were independently extracted by two different reviewers (VE, MB), and eventual initial disagreements were solved by further review and discussion among them.

### 2.4. Quality Assessment of Diagnostic Accuracy Studies

The QUADAS (Quality Assessment of Diagnostic Accuracy Studies) tool was applied to assess study quality and to evaluate risk of bias and concerns regarding applicability [13]. The risk of bias and concerns regarding applicability for every study were evaluated by two coauthors (VE, MB). The risk of bias included the four domains “patient selection”, “index test”, “reference standard”, and “flow and timing”, and the section regarding applicability included the three domains “patient selection”, “index test”, and “reference standard”. Answering different signaling questions specific for this review, each category was ranked as high, low, or unclear, respectively.

## 3. Results

### 3.1. Study Selection

**Figure 1:**
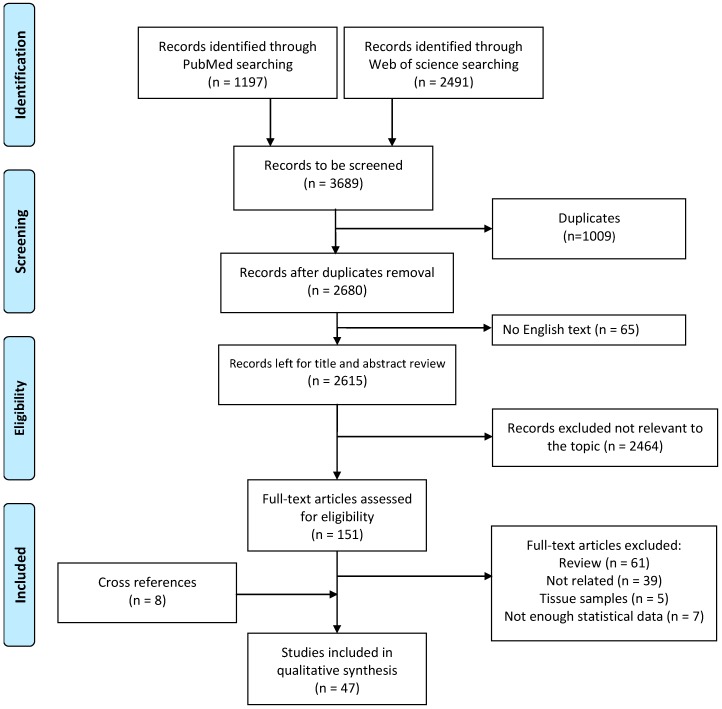
Preferred Reporting Items for Systematic Reviews and Meta-Analyses (PRISMA) Flow Diagram for systematic literature research using the PubMed and Web of Science databases.

We conducted a systematic literature research and retrieved 1197 records in the PubMed database and 2491 articles in Web of Science. The workflow of study selection and exclusion followed the PRISMA guidelines (Figure 1). After removal of duplicates (*n* = 1009) and articles that were not available in English (*n* = 65), the remaining 2680 articles were screened through title and abstract. After exclusion of non-eligible papers, 151 articles were left for careful full-text screening. Full text articles were further excluded if they were reviews or not related to the topic, if they were studies on tissue samples, or did not report enough statistical data on diagnostic performance. In total, 39 full text articles were eligible and an additional 8 articles were included as cross references. In summary, 47 original articles were considered in this systematic review.

### 3.2. Study Design and Population Characteristics

Table 1 gives an overview of study design and population characteristics of the 47 studies on metabolomics-based biomarkers for early detection of CRC and advanced adenomas. Out of these, 27 studies reported on blood-based biomarkers (17 serum, 9 plasma, and 1 dried blood spot), 16 on urinary markers, and 4 on fecal biomarkers. Most of the included articles presented a case-control study design (40 studies), and the majority of the studies were conducted in an Asian population (32 studies). Technologies used were mass spectrometry (MS, 37 studies), nuclear magnetic resonance (NMR) spectroscopy (8 studies), enzyme linked immune-sorbent assay (ELISA, 1 study), and an enzymatic assay (1 study). The numbers of cases ranged from 320 CRC cases [14] to 11 CRC cases in the smallest study [15], and the number of controls ranged from 633 healthy controls in a screening setting [16] to 10 controls in the smallest studies [15,17]. Age ranged from 22 to 93 years among the CRC cases and from 18 to 95 years among controls. 

Whenever possible, performance characteristics were extracted with a healthy control group as the reference group. One study only used diseased controls [18], and some studies additionally combined healthy individuals with people with benign colorectal diseases [19,20,21,22]. Uchiyama et al. combined carriers of adenomas with healthy controls to distinguish from CRC cases but reported on characteristics to distinguish adenomas from healthy controls as well [23]. Performance characteristics of metabolites and panels for specific study population subgroups are presented in Supplementary Table S1 and Supplementary Table S2.

### 3.3. Diagnostic Performance of Potential Biomarkers

Potential biomarkers for early detection of CRC were found in different bio fluidic sample types (blood, urine, feces) and vary in their biochemical classes. Most of the included studies (35 out of 47) used a panel of metabolites to discriminate diseased from control participants; a few reported only on performance characteristics for single metabolites (12 studies), but the composition of the panels and potential markers differed (Table 2, Supplementary Table S3). Internal validation was performed by subsampling, bootstrapping, or cross-validation in 25 studies. 

**Figure 2:**
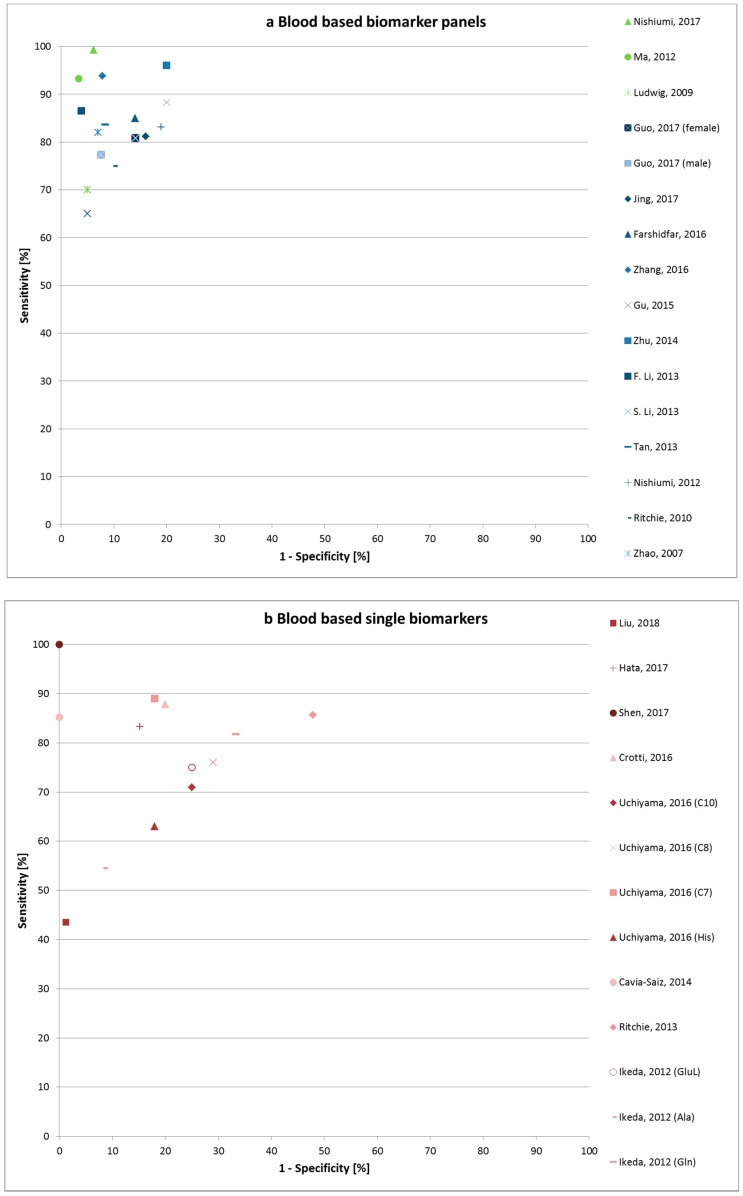
Sensitivity and 1-specificity of blood-based metabolic biomarker panels (**a**) and single biomarkers (**b**). In (**a**), not validates biomarker panels are marked in green, and (internally) validated panels are marked in blue color. Abbreviations: Ala, alanine; C7, benzoic acid; C8, octanoic acid; C10, decanoic acid; Gln, glutamine; GluL, glucuronic lactone; His, histidine.

For the blood-based markers, 14 (out of 27) studies were internally validated. Blood-based markers can be found either in serum or plasma samples or in dried blood spots. The latter methodology has some advantages, as smaller blood volumes are needed, no immediate processing is required, and transport and storage are very easy [18]. The biomarker pattern investigated by dried blood spots consisted of 4 amino acids and 4 acylcarnitines and showed good performance characteristics with 81.2% sensitivity and 84.0% specificity [18]. However, the majority of CRC patients in this study (53 out of 85, 62%) were in an advanced stage (III or IV) of the disease. The apparent best performance characteristics for blood based panels were reported in a study from Nishiumi et al. [39] for a combination of 8 metabolites (99.3% sensitivity, 93.8% specificity, and AUC 0.996) to differentiate early stages from healthy controls, but the pattern was not validated (Figure 2a,b). The highest sensitivity and specificity were reported for a single marker, but the study population was small, healthy controls were young (18–22 years), and no validation was performed [40]. Hata et al. (2017) and Ritchie et al. (2013) both found gastrointestinal tract acid 446 (GTA-446) to be a promising new biomarker with sensitivity of 83.3% and specificity of 84.8%, 85.7%, and 52.1%, respectively [25,30]. Decanoic acid was also found to be a promising biomarker candidate according to two independent studies with good characteristics (sensitivity 87.87%, specificity 80.0%, 71.0%, and 75.0%, respectively) [23,41].

**Figure 3:**
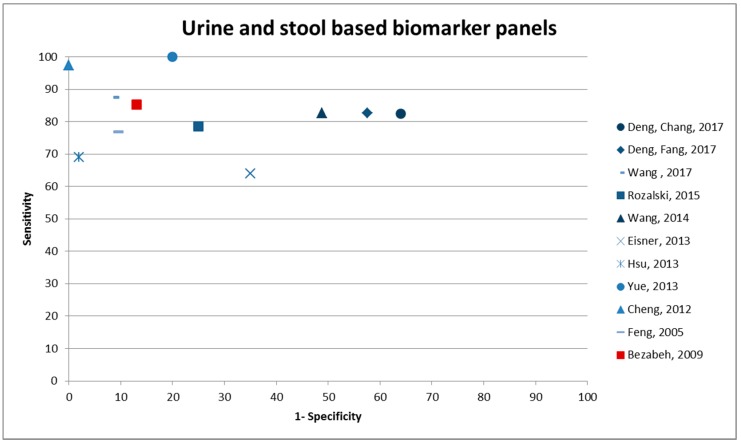
Sensitivity and 1-specificity of urine and stool-based metabolic biomarker panels. Urine based biomarker panels are presented in blue; the only stool based panel reporting on sensitivity and specificity is shown in red.

The majority of the studies investigating urinary biomarkers found a panel to be more appropriate than single metabolites (14 patterns, 2 single metabolites). The results from three Canadian papers are based on the same study setting [16,48,51]. The study with the highest sensitivity included 10 different metabolites, of which one was unknown and six metabolites were included in which the chemical formula (confirmed by MS) was known but structures were not further classified [17]. Performance characteristics were internally validated by subsampling, and sensitivity was 100% at 80.0% specificity, but samples sizes were low. The highest specificity (100.0%) was reported for a cross-validated panel of seven metabolites with 97.5% sensitivity (AUC 0.998) [54]. Deng, Fang et al. (2017) validated a biomarker panel that was suggested by H. Wang et al. in 2014, and they found similarly high sensitivity [48,51]. N1, N12-Diacetylspermine was found to be an individual biomarker candidate by two different studies [47,56]. Performance indicators of urine and stool-based biomarker panels can be found in Figure 3.

Biomarkers in stool samples for early detection of colorectal neoplasms were all internally validated. One study based on a three metabolite panel reported an AUC of 1.0 [15], but population size was very small (11 CRC cases and 10 controls). Another metabolomics panel found among participants of a true screening study was able to detect advanced colorectal neoplasms with good performance characteristics (AUC 0.94) [59]. 

Table 3 summarizes results for metabolites that were assessed three times or more often in combination as potential markers in blood samples. Some studies focused primarily on amino acids [27,33,45] or on fatty acids and other lipid derivatives [25,26,29,30,36,41,43,46]. Some metabolites, e.g., arginine, histidine, or tyrosine, were consistently found to be downregulated in blood samples from CRC patients compared to those from healthy controls, but results from other metabolites are not as clear and further research is needed. The metabolites, which were most identified as promising biomarkers in urine samples, were nucleosides (Table 4). The nucleoside concentration in the urine of CRC cases was higher compared to controls, and, consequently, urinary excretion of nucleosides is increased in diseased status. The most often identified metabolites in stool samples were glutamate/glutamic acid and butyrate/butyric acid, which were detected to be significantly different [58,59,60] in cases such as participants with CRC or advanced colorectal neoplasms, compared to healthy individuals (Table 5). Excretion of glutamine and glucose in CRC stool samples was reported to be decreased, but results on the other metabolites are not consistent regarding their deregulation. 

### 3.4. Quality Assessment of Diagnostic Accuracy Studies

We assessed risk of bias and concerns regarding applicability using the QUADAS-2 tool. The results are presented in Supplementary Table S4, and an overview is presented in Supplementary Figure S1. The risk of bias for the ‘patient selection’ section was high in 38 out of the 47 included studies, as most of the studies used a case-control study rather than screening cohort designs. However, the risk was low for ‘index test’ in 25 out of 47 studies. Many studies accounted for the pre-analytical validity, but validation, especially external, is often missing. The risk of bias for the ‘reference test’ was often rated as ‘unclear’, as it is often not reported clearly if the healthy controls underwent any form of endoscopy to ensure a healthy bowel status. The risk of bias for ‘flow and timing’ was low for 21 (out of 47) and unknown for the remaining studies. It is favorable when bio-fluids are collected before a reference standard is conducted. There are only minor concerns regarding applicability for the ‘index test’, as these index tests match our review question. In the section ‘patient selection’, concerns regarding applicability were high for the majority (39 out of 47 studies). Again for the ‘reference standard’, concerns regarding applicability were low for the most studies or unclear. 

## 4. Discussion

In this systematic review, we identified a large number of studies focusing on single metabolomic biomarkers or biomarker panels for detection of colorectal neoplasms, some of which reported good diagnostic performance characteristics. Most of the included studies were conducted in Asian countries and had a case-control study design. A MS-based approach with various modifications was the most frequently used platform. Generally, better diagnostic performance was reported for biomarker panels than for single biomarkers. Although the included studies report that different metabolite panels have best diagnostic performance characteristics, some consistency with respect to certain metabolites could be identified. Most of the studies focused on amino acids in blood samples and on nucleosides in urine samples as promising biomarker candidates. However, most of the findings lack a reliable form of validation.

### 4.1. Metabolomic Biomarkers of Cancer

Metabolomics is a promising approach for cancer detection, since cancer can be considered a metabolic disease and, so far, only few metabolic pathways seem to be altered during cancer state, which are aerobic glycolysis, glutaminolysis, and one-carbon metabolism [61]. Metabolomics represents downstream products in the cellular cascade and an integration of different approaches; for example, metabolomics with proteomics might be useful [62] and improved AUC values were shown when protein and metabolite biomarkers were combined, whereas the well-known CEA marker only had moderate performance when used as a single marker [63]. 

### 4.2. Influences on Metabolomics Profiles

Metabolites are closely related to the phenotype representing the processes in an organism. However, the metabolic profile is not a status but more a dynamic picture changing with the influence of the host itself or the environment, diet, or lifestyle factors [7]. Urine samples were more affected by diet than serum samples [64]. It could be shown that different types of diet affect the urinary metabolite composition [65]. However, it is estimated that diet plays only a minor part in changes of serum metabolites, and there are other factors contributing more to the variation such as gut microbiota composition [66]. It could be shown that the gut microbiota is different in patients with CRC compared to healthy controls [59,67] and is directly involved in carcinoma development [68,69]. The differences in the microbiota among diseased individuals and healthy controls might be responsible for differences in the metabolome of stool samples between CRC cases and healthy people, as bacteria are involved for example in metabolism of short-chain fatty acids [68]. It could be shown that the microbiota composition may be useful to distinguish even adenoma cases from healthy controls [70]. 

Other major confounding factors are lifestyle factors like smoking and physical activity. It was shown that various metabolites in blood samples were associated with the smoking status and number of cigarettes consumed per day [71]. Moreover, another study has shown that tobacco has influence on the metabolic profile, besides being directly associated with elevated risk of CRC [72]. Smoking itself is a well-known major risk factor for CRC [73,74]. Dependent upon the type and intensity of exercise and training status, physical activity which is associated with reduced risk of developing colorectal neoplasms [75,76] also influences the metabolite profile of blood and urine [77]. 

Controlling and reporting on potentially influencing factors is essential to reduce confounding variables [78]. Factors such as gender and age have an influence on body metabolite composition [79]. Next to these biological factors, time of sample collection is important because of the variation by the circadian rhythm [80]. In contrast to urine, serum metabolite profiles show less diurnal variation and less inter- and intra-subject variability [7]. Metabolite measurement is challenging because of the heterogeneity of the biochemical classes. Therefore, it is not possible to measure all metabolites with a single method. Different MS-based or NMR spectroscopy-based methods are used to enable the detection of a broad metabolite spectrum [7]. However, a good agreement between most laboratories in their performance of the methods in a targeted MS-approach was seen [81]. Other used technologies such as conventional ELISA assay can mostly assess one substrate at a time but are able to quantitatively assess the analytes. New multiplex assays enable detection of several substrates at a time [82].

Technical aspects, such as pre-analytics, have a great influence on the measured metabolic profile. An essential part is the time frame and temperature between sample collection and freezing. It was shown for urine samples that a full day storing at room temperature or on cool packs altered metabolite concentration, and that more than 2 freeze and thaw cycles affected the metabolic profile significantly [83]. Blood samples show a different picture. Previous freeze-and-thaw experiments indicate sufficient stability for the majority of the metabolites [84,85,86]. Metabolites in serum remained stable over a 4-months period frozen at −80 °C [87]. The biological reproducibility was good in plasma samples for the majority of metabolites over a 1-year period after storage in liquid nitrogen [88]. However, storage at room temperatures affected the blood metabolic profile, as well as urinary metabolites [84]. As handling aspects influence the composition of the metabolome, it is important to standardize protocols on sample collection, pre-analytical sample handling, and storage conditions to keep variations as low as possible. In particular, measures to ensure identical pre-analytics for cases and controls are indispensable for valid evaluation of diagnostic performance.

### 4.3. Comparison of Blood versus Urine

Blood and urine are “easily accessible” body fluids representing the systemic metabolomics profile. A limitation of these systemic samples compared to tissue samples is that the solid tumor itself is not directly analyzed. Cells and cell components leaking into the peripheral fluids and organs lead to a dilution of the target analytes in addition to other non-tumor components that can be found in the fluids [89]. Analysis of blood can be more complex than of urine, as urine contains fewer proteins, and high abundant proteins must be depleted from blood prior to analysis [90]. However, as urine is more affected by day–night cycles or diet, collection time is critical and correct documentation essential [91]. Blood is the primary carrier of circulating metabolites in the body, and both serum and plasma are considered for early detection analysis depending on the technology chosen [91]. As serum samples contain higher concentrations of metabolites, investigation of serum samples show more positive results than plasma sample investigations which demands even more careful validation of the results [85,92]. The composition of plasma and serum metabolites appears to be very similar, but some metabolites, for example eicosanoids, increase during the clotting process in serum [93]. 

### 4.4. Limitations

There are several limitations that make the interpretation and implementation of metabolomics studies difficult. An issue of particular concern is the lack of standardization [94]. The Standard Metabolomics Reporting Structures (SMRS) group tried to standardize protocols for metabolomics studies beginning with study design, sample collection, and preparation to ensure their application in the future [95]. The lack of standardization might limit the comparability of the included studies in this systematic review. 

Another limitation is the lack of independent validation of the biomarkers in controlled clinical settings or, even better, in a true screening cohort in asymptomatic people for early detection of cancer [96]. Most of the studies report no validation of their biomarker panel. The lack of validation may often result in overestimation of the performance of biomarker panels due to overfitting. In other studies, only internal validation was used, in which case generalizability remains an open issue of potential concern. Most of the studies are conducted in relatively small sample sizes, limiting the power for discovery of valid biomarkers with adequate control for multiple testing [94]. Before the implementation of metabolomics for early detection in clinical practice, major efforts are needed to set up true screening cohorts with large population sizes under standardized conditions. Moreover, the majority of the studies were conducted among Asian populations, which may limit the generalizability and transferability to other ethnic groups.

Besides limitations of the studies included in this review, this systematic review may be limited by publication bias, less than perfect identification of relevant studies, and lack of detail and heterogeneity of information provided by the individual study publications.

## 5. Conclusions

Deaths from colorectal cancer could be mostly prevented by early detection and treatment of the cancer and its precursors. Although effective screening offers have been established, adherence to these offers remains limited due to their invasiveness (e.g., colonoscopy) or due to their being based on collection of stool samples (e.g., fecal immunochemical tests for hemoglobin). Blood or urine-based tests could be an attractive alternative if they were able to detect colorectal cancer and its precursors with good diagnostic performance. Metabolomics approaches are promising, as they are closely related to the phenotype, which means to directly detectable effects and changes in a biological system. A panel of metabolites seems to be more promising for use as biomarkers for advanced colorectal neoplasms than a single marker. We discovered consistency in findings with regards to amino acids in blood samples and nucleosides in urinary samples. Still, heterogeneous results demand more research on that topic before metabolomics biomarkers are ready for use as screening biomarkers in clinical settings. In particular, larger studies conducted in true screening settings and external validation of the findings are needed. To further improve diagnostic performance of non-invasive tests for early detection of CRC or its precursors, the combination of different approaches such as metabolomics and proteomics should be considered.

## Figures and Tables

**Table 1:** Study characteristics.

Characteristics of the Studies				Training Set (if Applicable)				Validation Set (if Applicable)			
	First Author, Year	Study Type Country	Study Group	Population *n*	Age (Range/SD)	Male (%)	Stage (0)/I/II/III/IV/(?)	Population *n*	Age (Range/SD)	Male (%)	Stage (0)/I/II/III/IV/(?)
	Dried blood spot										
1	Jing, 2017 [18]	Case-control Japan	CRC P	85 81	61.0 (22–92) 57.9 (29–79)	59 58	10/22/31/22				
	Serum										
2	Zhang, 2018 [22]	Case-control China	CRC Enteritis Cn	55 34 52	63.5 (±4.2) 56.9 (±8.2) 60.8 (±6.4)	56 56 38	n.a.				
3	Guo, 2017 [24]	Case-control China	CRC Cn	144 144	62 ± 11/63 ± 9 62 ± 11/63 ± 10	46 46	I + II/III + IV/(?) 58/77/(9)				
4	Hata, 2017 [25]	Case-control China	CRC Cn	225 916	n.a. n.a.	60 62	(21)/70/49/71/13/(1)				
5	Uchiyama, 2017 [23]	Case-control Japan	CRC A Cn	56 59 60	70.4 ^1^ 69.9 (±8.2) 67.7 (±9.2)	50 51 50	14/14/14/14				
6	Farshidfar, 2016 [14]	Case-control Canada	CRC A Cn	320 31 254	n.a. 59.5 (±6.0) 61.7 (±9.3)	n.a. 68 58	47/60/71/142				
7	Zhang, 2016 [26]	Case-control China	CRC BCD Cn	59 0 69	59.1 (±11.4) n.a. 57.9 (±10.4)	58 n.a. 52	1/3/23/15	80 55 116	59.5 (±10.3) 58.2 (±10.9) 58.9 (±10.4)	45 62 62	21/14/23/14
8	Gu, 2015 [27]	Case-control USA	CRC Cn	28 28	56 ^med^ (29–88) 58 ^med^ (18–80)	50 50	1/2/6/19				
9	Zhu, 2014 [28]	Case-control USA	CRC P Cn	66 76 92	58 ^med^ (27–88) 56 ^med^ (37–86) 57 ^med^ (18–80)	45 49 49	I + II/III/IV 21/17/28				
10	F. Li, 2013 [29]	Case-control China	CRC Cn	52 52	56 ^med^ (24–91) 52 ^med^ (22–88)	54 54	Early/late 26/26				
11	Ritchie, 2013 [30]	Screening Canada	CRC Cn	98 4825	57 ^med^ (18–92) ^2^	45 ^2^	30/22/34/12				
12	Tan, 2013 [31]	Case-control China	CRC Cn	62 62	60.1 (24–82) 59.4 (31–75)	55 45	16/25/17/4	39 40	61.8 (36–80) 55.9 (35–76)	59 0	10/18/9/2
13	Ikeda, 2012 [32]	Case-control Japan	CRC Cn	12 12	71.3 ^med^ (63–83) 58.5 ^med^ (45–74)	67 42	3/4/5/0				
14	Leichtle, 2012 [33]	Case-control Germany	CRC Cn	59 58	59 ^med^ (45–90) 58 ^med^ (38–75)	63 45	5/18/20/16				
15	Ma, 2012 [34]	Case-control China	CRC Cn	30 30	65.03 ^mean^ (53–72) 64.97 ^mean^ (53–72)	60 60	3/13/8/6				
16	Nishiumi, 2012 [35]	Case-control Japan	CRC Cn	60 60	67.7 ^mean^ (36–88) 64.5 ^mean^ (39–88)	65 65	(12)/12/12/12/12	59 63	64.8 ^mean^ (31–84) 62.8 ^mean^ (47–73)	51 51	(15)/11/3/11/19
17	Ritchie, 2010 [36]	Case-control Japan, USA	CRC Cn	112 110	62.0 (28–90) ^3^ n.a.	56 36	23/38/35/11/(5)	110 110	69.2 (35–91) 3 55.8 (26–86) 3	57 59	0+I/II/III/IV/(?) 22/39/36/9/(4)
18	Ludwig, 2009 [37]	Case-control UK	CRC +A Cn	38 19	67 (±13) 63 (±10)	n.a. n.a.	A + B/C + D 18/20 (+ 8 A)				
	Plasma										
19	Liu, 2018 [38]	Case-control China	RC A Cn	155 85 80	57.0 (±11.8) 55.0 (±10.9) 51.2 (±12.5)	51 26 24	32/38/50/35				
20	Nishiumi, 2017 [39]	Case-control Japan	CRC Cn	282 291	67.0 ^mean^ (40–93) 66.8 ^mean^ (41–88)	60 61	(79)/80/123/0/0				
21	Shen, 2017 [40]	Case-control China	CRC Cn	25 10	n.a. (31–80) n.a. (18–22)	64 50	n.a.				
22	Crotti, 2016 [41]	Case-control Italy	CRC Cn	48 20	67 (49–90) 62 (35–83)	56 50	11/9/16/12				
23	Cavia-Saiz, 2014 [42]	Case-control Spain	CRC Cn	78 ^4^ 70	n.a. n.a.	69 n.a.	I + II/III/IV 11/24/43/4				
24	S. Li, 2013 [43]	Case-control China	CRC AP Cn	120 120 120	55.7 (±11.8) 54.5 (±14.2) 55.7 (±7.5)	59 63 68	I + II/III + IV/(?) 15/93/(12)				
25	Miyagi, 2011 [44]	Case-control Japan	CRC P Cn	199 34 995	63.7 (±9.5) 55.3 (±7.9) 62.4 (±9.5)	57 21 57	(8)/63/48/59/19/(2)				
26	Okamoto, 2009 [45]	Case-control Japan	CRC Cn	49 49	64.1 (40-78) 59.6 (40–69)	78 78	(2)/7/19/14/6/(1)	13 54	57.5 (33–75) 55.8 (40–69)	31 26	2/3/8/0
27	Zhao, 2007 [46]	Case-control USA	CRC Cn	89 83	62.0 (±14.1) 46.3 (±15.4)	64 45	I + II/III + IV/(?) 37/49/(3)	44 42	62.9 (±10.5) 45.4 (±16.6)	70 48	I+II/III+IV/(?) 16/26/(1)
	Urine										
28	Nakajima, 2018 [47]	Case-control Japan	CRC Benign Cn	201 14 17	68.7 (±0.8) 65.0 (±3.1) 42.1 (±2.8)	58 79 76	(1)/27/28/109/34 Tis 2				
29	Deng, Chang, 2017 [48]	Screening Canada	CRC/A Cn + HPP	1/154 530	59.9 ^mean^ (±7.4) 56.1 ^mean^ (±8.2)	61 42	n.a.				
30	Deng, Fang, 2017 [19]	Screening China	A Cn	345 316	65.1 ^mean^ (±6.6) 61.8 ^mean^ (±7.4)	57 26	n.a.				
31	Wang, 2017 [49]	Case-control China	CRC Cn	55 40	n.a. (27-84) 59 (28-78)	47 48	I + II/III + IV 23/32				
32	Rozalski, 2015 [50]	Case-control Poland	CRC A Cn	56 15 72	65 ^med^ 66 ^med^ 54 ^med^	58 53 41	n.a.				
33	Wang, 2014 [51]	Screening Canada	A Cn	162 422	59.1 (±0.6) 55.7 (±0.4)	59 43	n.a.	81 211	60.4 (±0.8) 56.1 (±0.6)	62 42	n.a.
34	Eisner, 2013 [16]	Screening Canada	HPP/A/ CRC Cn	110/243/2 633	58.9 ^mean^ (±8.2) 56.2 ^mean^ (±8.1)	55 42	n.a.				
35	Hsu, 2013 [52]	Case-control China	CRC Cn	26 45	65.3 (±14.0) n.a.	46 n.a.	3/6/10/7				
36	Yue, 2013 [17]	Case-control China	CRC Cn	29 10	n.a. n.a.	n.a. n.a.	n.a.				
37	Chen, 2012 [53]	Case-control China	CRC Cn	20 14	n.a. (37–87) 68 med (50–86)	50 57	I + II/III + IV 8/12				
38	Cheng, 2012 [54]	Case-control China	CRC Cn	61 62	59 ^med^ (24–83) 60 ^med^ (31–75)	58 50	15/25/17/4	40 41	63.5 ^med^ (36–80) 57 ^med^ (35–76)	60 0	9/20/10/1
39	Wang, 2010 [21]	Case-control China	CRC BCT Cn	50 34 34	n.a. n.a. n.a.	n.a. n.a. n.a.	n.a.				
40	Johnson, 2006 [20]	Case-control USA	CRC BCD Cn	58 28 72	60.9 (±10.0) 38.8 (±11.7) 60.9 (±7.5)	55 46 74	n.a.				
41	Feng, 2005 [55]	Case-control China	CRC Cn	52 62	63 ^med^ (26–87) 59 ^med^ (24–78)	52 53	A/B/C/D 5/22/18/7				
42	Hiramatsu, 2005 [56]	Case-control Japan	CRC BGD Cn	248 51 52	n.a. n.a. (22–52)	n.a. n.a. 52	(20)/40/60/107/21				
43	Zheng, 2005 [57]	Case-control China	CRC A Cn	52 10 60	60.0 ^med^ (26–87) n.a. 52 ^med^ (21–71)	56 n.a. 52	7/23/15/7				
	Feces										
44	Lin, 2016 [58]	Case-control China	CRC Cn	68 32	56 (±21) 57 (±23)	53 47	I + II/III/IV 20/2523				
45	Amiot, 2015 [59]	Cohort France	ACN Cn	33 22	59.4 ^med^ (±6.9) 52.0 ^med^ (±12.0)	76 68	n.a.				
46	Phua, 2014 [15]	Case-control China	CRC Cn	11 10	64.5 ^mean^ (56–80) 57.4 ^mean^ (48–79)	64 40	A/B/C/D 0/6/5/0				
47	Bezabeh, 2009 [60]	Screening China	CRC Cn	111 412	n.a. n.a.	n.a. n.a.	n.a. n.a.				

Abbreviations: (A)A, (advanced) adenoma; ACN, advanced colorectal neoplasia; AP, adenomatous polyps; BCD, benign colorectal disease; BCT, benign colorectal tumor; BGD, benign gastrointestinal disease; Cn, controls; CRC, colorectal cancer; HPP, hyperplastic polyp; ^med^, median; P, polyps; RC, rectal cancer; Tis, tumor in situ. ^1^ Mean age calculated from available subgroup data. ^2^ The numbers account for the whole study population without distinguishing between cases and controls. ^3^ Training set participants from Genomics Collaborative, Seracare 1, and Osaka participants, validation set from Chiba and Seracare 2 study. Mean age calculated from available subgroup data. ^4^ Inconsistency in reporting the numbers of included CRC patients.

**Table 2:** Performance characteristics of single metabolites and panels of potential biomarkers.

First Author, Year		Metabolites				Diagnostic Performance							
	Outcomes	Am A/	FA	CH	Others	Sn	Sp	AUC-No	AUC with Validation				*p*-Value
		Pep						Validation	SS	CV	BS	EV	
Biomarker panels													
Dried blood spot													
Jing, 2017 [18]	CRC	4	4	0	0	81.2	84.0			0.91			<0.05
Serum													
Zhang, 2018 [22]	CRC	0	2	0	0	n.a.	n.a.	0.90					<0.05
Guo, 2017 [24]	CRC ♂ CRC ♀	0 0	5 2	0 0	0 0	77.3 80.8	92.4 85.9	0.90 0.90					n.a. n.a.
Farshidfar, 2016 [14]	CRC	9	7	12	13	85.0	86.0		0.91	0.91			<0.00001
Y. Zhang, 2016 [26]	CRC	0	6	0	0	93.8	92.2		0.98				<0.001
H. Gu, 2015 [27]	CRC	8	0	0	0	65.0	95.0			0.91			<0.05
Zhu, 2014 [28]	CRC	7	3	3	0	96.0	80.0		0.93	0.93 ^1^			<0.05
F. Li, 2013 [29]	CRC	0	9	0	0	86.5	96.2	0.96					<0.05
Tan, 2013 [31]	CRC	6	1	3	0	83.7	91.7	n.a.					<0.05
Ma, 2012 [34]	CRC	3	0	3	0	93.3 ^2^	96.7 ^2^	n.a.					<0.05
Nishiumi, 2012 [35]	CRC	3	0	1	0	83.1	81.0		n.a.				<0.05
Ritchie, 2010 [36]	CRC	0	3	0	0	75.0	90.0					0.91	<0.05
Ludwig, 2009 [37]	CRC	0	1	4	0	70.0	95.0	n.a.					n.a.
Plasma													
Nishiumi, 2017 [39]	Stage 0/I/II	3	3	2	0	99.3	93.8	1.00					0.000781
S. Li, 2013 [43]	CRC	0	3	0	0	88.3	80.0				n.a.		<0.05
Miyagi, 2011 [44]	CRC	10	0	0	0	n.a.	n.a.			0.87 ^3^			<0.001
Okamoto, 2009 [45]	CRC	6	0	0	0	n.a.	n.a.		0.91				<0.05
Zhao, 2007 [46]	CRC	0	4	0	0	82.0	93.0		n.a.				<0.001
Urine													
Nakajima, 2018 [47]	CRC	2	0	0	0	n.a.	n.a.				0.79		<0.0001
Deng, Chang, 2017 [48]	AP	0	1	2	0	82.4 ^4^	36.0 ^4^		0.69				<0.05
Deng, Fang, 2017 [19]	AP	7	2	8	0	82.6	42.4	0.72					n.a.
Wang, 2017 [49]	CRC I/II	3	0	1	0	87.5	91.3		0.93				<0.01
Rozalski, 2015 [50]	CRC	0	0	3	0	78.6	75.0	0.78					<0.0001
Wang, 2014 [51]	AP	7	2	8	0	82.7	51.2		n.a.	n.a.			<0.05
Eisner, 2013 [16]	P	2	0	2	0	64.0	65.0			0.72			<0.01
Hsu, 2013 [52]	CRC	0	0	6	0	69.0	98.0	n.a.					<0.01
Yue, 2013 [17]	CRC	0	9	0	1	100.0	80.0		n.a.				<0.05
Chen, 2012 [53]	CRC	8	0	4	0	n.a.	n.a.	1.00					<0.01
Cheng, 2012 [54]	CRC	4	1	2	0	97.5	100.0		1.00	1.00			<0.001
Wang, 2010 [21]	CRC	4 0	5 0	0 7	0 0	n.a. n.a	n.a n.a			0.96 0.89			<0.05 <0.05
Feng, 2005 [55]	CRC	0	0	2	0	71.2	93.3	n.a.					<0.01
Zheng, 2005 [57]	CRC	0	0	14	0	71.0	96.0	n.a.					<0.05
Feces													
Amiot, 2015 [59]	ACN	2	4	1	0	n.a.	n.a.			0.94			<0.0001
Phua, 2014 [15]	CRC	0	1	2	0	n.a.	n.a.			1.00			<0.05
Bezabeh, 2009 [60]	CRC	3	2	0	0	85.2	86.9		0.92	0.92 ^3^			n.a.
Single markers													
Serum													
Hata, 2017 [25]	CRC	0	1	0	0	83.3	84.8	0.91					<0.05
Uchiyama, 2017 [23]	CRC	0 0 0 1 ^His^	1 ^C7^ 1 ^C8^ 1 ^C10^ 0	0 0 0 0	0 0 0 0	89.0 76.0 71.0 63.0	82.0 71.0 75.0 82.0	0.89 0.83 0.79 0.74					<0.01 <0.01 <0.01 <0.01
Ritchie, 2013 [30]	CRC	0	1	0	0	85.7	~52.1 ^5^	n.a.					<0.05
Ikeda, 2012 [32]	CRC	1 ^Ala^ 0 1 ^Gln^	0 0 0	0 1 ^GluL^ 0	0 0 0	54.5 75.0 81.8	91.6 75.0 66.7	n.a.					<0.05
Leichtle, 2012 [33]	CRC	1	0	0	0	n.a.	n.a.				0.71		<0.001
Plasma													
Liu, 2018 [38]	RC/A	1	0	0	0	43.5	98.8	0.71					<0.05
Shen, 2017 [40]	CRC	0 0	1 ^PG^ 1 ^SM^	0 0	0 0	1.00 1.00	1.00 1.00	1.00 1.00					<0.05 <0.05
Crotti, 2016 [41]	CRC	0	1	0	0	87.8	80.0	0.82					<0.01
Cavia-Saiz, 2014 [42]	CRC	1	0	0	0	85.2	100.0	0.92					<0.001
Urine													
Johnson, 2006 [20]	CRC	0	1	0	0	90.0	45.0	0.64					<0.05
Hiramatsu, 2005 [56]	CRC	1	0	0	0	75.8	96.0	n.a.					<0.0001
Feces													
Lin, 2016 [58]	Early stage	0 0	1 ^Ace^ 1 ^Suc^	0 0	0 0	94.7 91.2	92.3 93.5		0.99 0.94	0.99 0.94			<0.001 <0.001

The numbers in the column of the metabolites indicate how many metabolites were used for the biomarker panel from each biochemical subclass. In case of single markers, the biochemical subclass of the marker is listed. Abbreviations: (A)A, (advanced) adenomas; ^Ace^, acetate; ACN, advanced colorectal neoplasms; ^Ala^, alanine; Am A, amino acids, AP, adenomatous polyps; AUC, area under the curve; BS, bootstrapping; C7, benzoic acid; C8, octanoic acid; C10, decanoic acid; CH, carbohydrates; CV, cross validation; EV, external validation; FA, fatty acids; ^Gln^, glutamine; ^GluL^, glucuronic lactone; ^His^, histidine; LOOCV, leave one out cross validation; MCCV, Monte Carlo cross validation; P, polyps; pep, peptides; ^PG^, phosphatidylglycerol (34:0); RC, rectal cancer; ^SM^, sphingomyelin (38:8); Sn, sensitivity; Sp, specificity; SS, subsampling; ^Suc^, succinate. ^1^ Monte Carlo cross validation (MCCV). ^2^ Sensitivity and specificity calculated from available data. ^3^ Leave-one-out cross validation (LOOCV). ^4^ Additional results for different cut-off values can be read from the original article. ^5^ Specificity was calculated for the intended to screening population (40–74 years olds in the colonoscopy population).

**Table 3:** Metabolites assessed three times or more across different publications on blood biomarkers.

First Author, Year	Amino Acids																	Carbo- Hydrates		Fatty Acids	
	Alanine	Arginine	Aspartate Aspartic acid	Glutamate Glutamic acid	Glutamine	Glycine	Histidine	Leucine Isoleucine	Lysine	Methionine	Ornithine	Phenylalanine	Proline /Hydroxyproline	(allo) Threonine Threonic acid	Tryptophan	Tyrosine	Valine	Lactate Lactic acid	Pyruvate Pyruvic acid	2/3-Hydroxy-butyrate 3-Hydroxy-butyric acid	18:2 LPC
Liu, 2018 [38]																					
Zhang, 2018 [22]																					
Guo, 2017 [24]																					
Hata, 2017 [25]																					
Jing, 2017 [18]	↓	↓										↑R				↑R	↓				
Nishiumi, 2017 [39]									↓		↑				↓				↑		
Uchiyama, 2017 [23]							↓														
Shen, 2017 [40]																					
Crotti, 2016 [41]																					
Farshidfar, 2016 [14]	↑					↑		↑	↑			↑		↑		↑		↑			
Zhang, 2016 [26]																					
Gu, 2015 [27]		↓	↑	↑	↓		↓		↓^R^							↓					
Cavia-Saiz, 2014 [42]																					
Zhu, 2014 [28]	↓					↓	↓			↓			↑								
F. Li, 2013 [29]																					↓
S. Li, 2013 [43]																					→
Tan, 2013 [31]				↓							↓	↓	↓		↓				↑		
Ikeda, 2012 [32]	↑			→	↑					→			→					→		→	
Leichtle, 2012 [33]	↓		↓			↓	↓	↓	↓	↓				↓		↓	↓				
Ma, 2012 [34]						↓								↓			↓			↑	
Nishiumi, 2012 [35]			↑																	↑	
Miyagi, 2011 [44]	↑	↓					↓	↑	↑						↓	↓	↓				
Ritchie, 2010 [36]																					
Ludwig, 2009 [37]																		→	→	→	
Okamoto, 2009 [45]				↑	↑								↑	↓			↓				
Zhao, 2007 [46]																					↓

Abbreviations: ↑, increased levels in cases compared to healthy individuals; ↓, decreased levels in cases compared to healthy individuals; →, significant differences between cases and healthy individuals (not reported if increased or decreased); R, ratio. Empty lines indicate that this specific metabolite was not investigated in the corresponding study.

**Table 4:** Metabolites assessed three times or more across different publications on urine biomarkers.

First Author, Year	Amino Acids				Carbohydrates					
	Histidine	Serine	Trigonelline	Tyrosine	Acetone	Cytidine	Methyladenosine	Methanol	2,2,-Methylguanosine	Pseudouridine
Nakajima, 2018 [47]										
Deng, Chang, 2017 [48]										
Deng, Fang, 2017 [19]	→	↓	→	→	→			→		
Wang, 2017 [49]										
Rozalski, 2015 [50]										
Wang, 2014 [51]	→	↓	→	→	→			→		
Eisner, 2013 [16]			↑	↑	↓			↓		
Hsu, 2013 [52]						↑	↑			
Yue, 2013 [17]										
Chen, 2012 [53]	↓	↓								
Cheng, 2012 [54]										
Wang, 2010 [21]						↑	↑		↑	↑
Johnson, 2006 [20]										
Feng, 2005 [55]										↑
Hiramatsu, 2005 [56]										
Zheng, 2005 [57]						↑	↑		↑	↑

Abbreviations: ↑, increased levels in cases compared to healthy individuals; ↓, decreased levels in cases compared to healthy individuals; →, significant differences between cases and healthy individuals (not reported if increased or decreased). Empty lines indicate that this specific metabolite was not investigated in the corresponding study.

**Table 5:** Metabolites assessed two times or more across different publications on fecal biomarkers.

First Author, Year	Amino Acids				CH	Fatty Acids		
	Glutamate Glutamic acid	Glutamine	Isoleucine	Valine	β-Glucose	Acetate	Butyrate Butyric acid	Propionate
**Lin, 2016 [58]**	↓	↓	↑	↓	↓	↓	↑	↑
**Amiot, 2015 [59]**	↓	↓			↓	↑	↓	↑
**Phua, 2014 [15]**								
**Bezabeh, 2009 [60]**	→		→	→			→	

Abbreviations: ↑, increased levels in cases compared to healthy individuals; ↓, decreased levels in cases compared to healthy individuals; →, significant differences between cases and healthy individuals (not reported if increased or decreased); CH, carbohydrates. Empty lines indicate that this specific metabolite was not investigated in the corresponding study.

## Supplementary Materials

The following are available online at http://www.mdpi.com/2072-6694/10/8/246/s1, Figure S1: QUADAS overview; Table S1: performance characteristics of single metabolites and metabolomics panels of potential biomarkers with additional outcomes; Table S2: additional potential biomarkers and biomarker panels for detection of adenomas, advanced colorectal neoplasms, polyps, or early stage CRC; Table S3: biochemical affiliations of potential biomarkers and biomarker panels; Table S4: QUADAS tool.
