# Supplementary material for: Metabolomics Biomarkers for Detection of Colorectal Neoplasms: A Systematic Review
Source: Cancers (Basel). 2018 Jul 27;10(8):246. doi: 10.3390/cancers10080246 (PMC6116151; doi:10.3390/cancers10080246)
Supplement: Supplementary file 1 [file cancers-10-00246-s001.pdf]

Supplementary Materials: Metabolomics Biomarkers for Detection of Colorectal Neoplasms: A Systematic Review

Vanessa Erben, Megha Bhardwaj, Petra Schrotz-King and Hermann Brenner

Figure S1. QUADAS Overview.

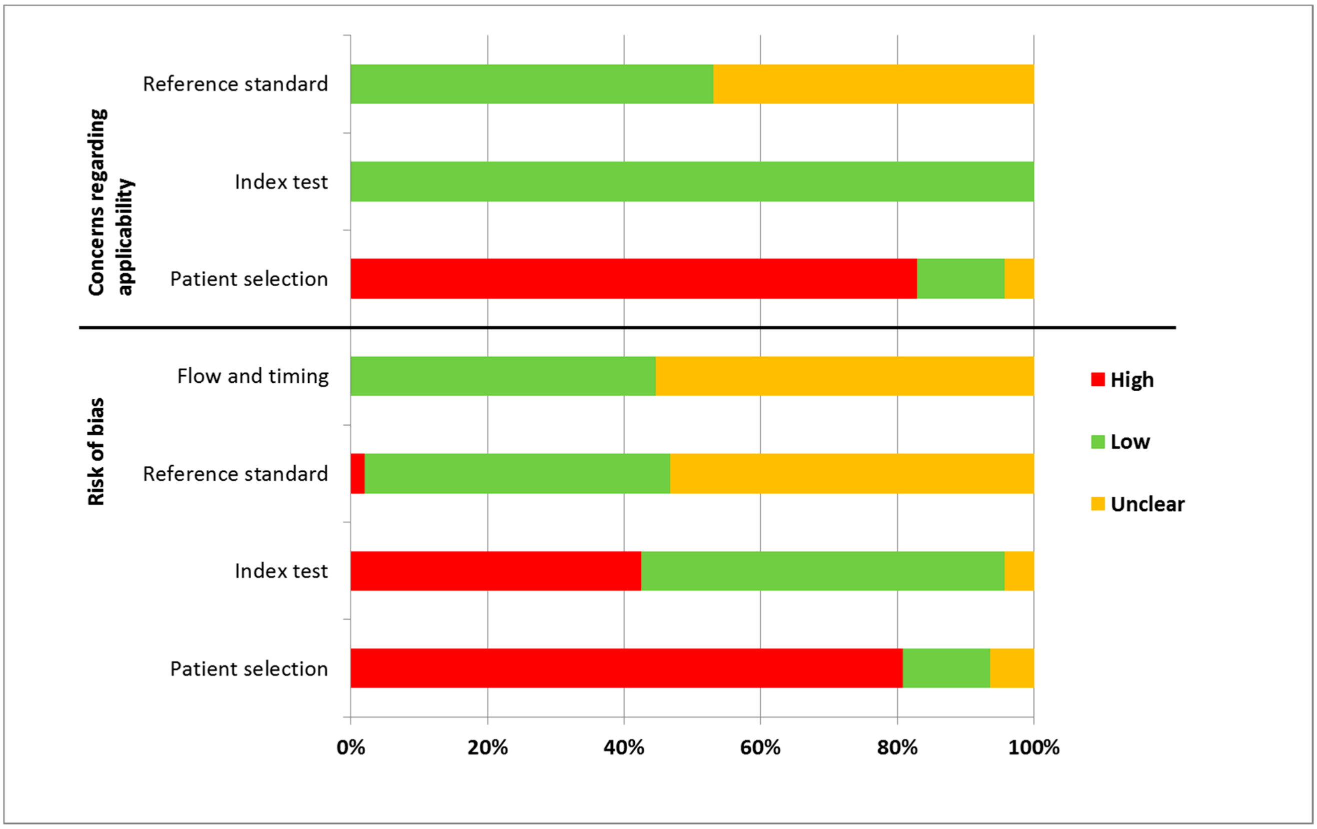

Note: red bar, proportion with high risk; green bar, proportion with low risk; yellow bar, proportion with unclear risk.

Table S1. Performance characteristics of single metabolites and metabolomic panels of potential biomarkers with additional outcomes.

First author, Year	Metabolites					Diagnostic performance							
	Outcomes	Am A/ Pep	FA	CH	Others	Sn	Sp	AUC - No validation	AUC with validation: SS	CV	BS	EV	p-value
**Serum**													
Guo, 2017 [24]	Stage I/II ♂	0	5	0	0	73.1	87.1	0.85					n.a.
	Stage I/II ♀	0	2	0	0	81.3	86.5	0.90					n.a.
Uchiyama, 2017 [23]	A	0	1 C7	0	0	88.0	85.0	0.92					<0.01
Farshidfar, 2016 [14]	A	2	2	1	9	n.a.	n.a.		0.81	0.81			<0.0001
Zhang, 2016 [26]	Early CRC vs. Cn + BCD	0	4	0	0	84.6	89.8		0.93				<0.001
F. Li, 2013 [29]	Stage I/II	0	10	0	0	98.1	100.0	0.99					<0.05
Nishiumi, 2012 [35]	Stage 0, I, I	3	0	1	0	82.8	n.a.		n.a.				<0.05
**Plasma**													
Nishiumi et al., 2017 [39]	Stage 0	3	3	2	0	100.0	100.0	n.a.					0.000781
	Stage I	3	3	2	0	97.5	91.3						
	Stage II	3	3	2	0	100.0	91.7						
S. Li, 2013 [43]	AP	0	4	0	0	89.0	80.0				n.a.		<0.05
Miyagi, 2011 [44]	Stage 0	10	0	0	0	n.a.	n.a.			0.90			<0.001
	Stage I	10	0	0	0					0.86			
	Stage II	10	0	0	0					0.92			
	Stage III	10	0	0	0					0.82			
	Stage IV	10	0	0	0					0.95			
**Urine**													
Nakajima, 2018 [47]	CRC vs. Cn + BCD	2	0	0	0	n.a.	n.a.				0.96		
Johnson, 2006 [20]	CRC/large P vs. small + no polyps	0	1	0	0	88.0	53.0	0.70					<0.05
Hiramatsu, 2005 [56]	Stage 0	1	0	0	0	60.0	n.a.	n.a.					<0.0001
	Stage I	1	0	0	0	62.5	n.a.						
	Stage II	1	0	0	0	71.7	n.a.						
	Stage III	1	0	0	0	83.2	n.a.						
	Stage IV	1	0	0	0	90.5	n.a.						

Abbreviations: (A)A, (advanced) adenomas; ACN, advanced colorectal neoplasms; Am A, amino acids, AP, adenomatous polyps; AUC, area under the curve; BCD, benign colorectal disease; BS, bootstrapping; C7, benzoic acid; CH, carbohydrates; CRC, colorectal cancer; CV, cross validation; EV, external validation; FA, fatty acids; P, polyps; pep, peptides; Sn, sensitivity; Sp, specificity; SS, subsampling.

Table S2. Additional potential biomarkers and biomarker panels for detection of adenomas, advanced colorectal neoplasms, polyps or early stage CRC.

Author, Year		Others
Serum		
Guo, 2017 [24]	♂Fatty acids: SM (34:1), PC (34:2), PC (34:1), PC (36:3), PC (36:2)<br>♀ Fatty acids: PC (36:4), PC (36:3)	
Uchiyama, 2017 [23]	Fatty acids: ↓ Benzoic acid	
Farshidfar, 2016 [14]	Amino acids/ peptides: → Cystine, Glu<br>Fatty acids: → heptadecanoic acid, glyceric acid<br>Carbohydrates: → erythriol	unknown (6), alkane (3)
Zhang, 2016 [26]	Fatty acids: ↓ C16:1 (palmitoleic acid), C18:2 (linoleic acid), C20:4 (arachidonic acid), C22:6 (docosahexanoic acid)	
F. Li, 2013 [29]	Fatty acids: ↓ Palmitic amide, oleamide , hexadecanedioic acid, octadecanoic acid, eicosatrienic acid, LPC(18:2), LPC(20:4), LPC(22:6), myristic [illegible]id, ↑ LPC(16:0)	
Nishiumi, 2012 [35]	Amino acids/ peptides: ↑ Asp, kynurenine, cystamine<br>Carbohydrates: ↑ 2-hydroxybutyrate	
Plasma		
Nishiumi, 2017 [39]	Amino acids/ peptides: ↑ Orn, ↓ Trp, Lys<br>Fatty acids: ↑ glycolic acid, ↓ palmitoleic acid, 3-hydroxyisovaleric acid<br>Carbohydrates: ↑ Pyruvic acid, fumaric acid	
S. Li, 2013 [43]	Fatty acids: →Saturated LPC, 20:4 LPC, 16:0 sphingomyelin, 18:0 sphingomyelin	
Miyagi, 2011 [44]	Amino acids/ peptides: ↑ Ser, Ala, Ile, Leu, Lys, ↓ Val, Tyr, His, Trp, Arg	
Urine		
Nakajima, 2018 [47]	Amino acids/ peptides: ↑ N1, N12-acetylspermine, N1,N8-acetylspermidine	
Johnson, 2006 [20]	Fatty acids: ↑ PGE-M	
Hiramatsu, 2005 [6]	Amino acids/ peptides: ↑ N1,N12-Diacetylspermine	

Abbreviations: Ala, alanine; Arg, arginine; His, histidine; Ile, isoleucine; Leu, leucine; LPC, lysophosphatidylcholine; Lys, lysine; Orn, ornithine; PGE-M, Prostaglandin E2 metabolite; Ser, serine, Trp, tryptophan; Tyr, tyrosine; Val, valine.

Table S3. Biochemical affiliations of potential biomarkers and biomarker panels.

First author, Year		Others
Dried blood spot		
Jing, 2017 [18]	Amino acids/ peptides: ↓ Arg, Phe/Tyr, Ala, Val<br>Fatty acids: ↑ C16, ↓ C4/C8, C5/C3, C4/C3	
Serum		
Zhang, 2018 [22]	Fatty acids: ↑ 5-hydroxyl-eicosatetraenoic acid, ↓ 12-hydroxyl-eicosatetraenoic acid	
Guo, 2017 [24]	Fatty acids ♂: SM (34:1), PC (34:2), PC (34:1), PC (36:3), PC (36:2); Fatty acids ♀: PC (36:4), PC (36:3)	
Hata, 2017 [25]	Fatty acids: ↓ Gastrointestinal tract acid 446 (GTA-446)	
Uchiyama, 2017 [23]	Amino acids/ peptides: ↓ His;<br>Fatty acids: ↑ Octanoic acid, decanoic acid, ↓ Benzoic acid	
Farshidfar, 2016 [14]	Amino acids/ peptides: ↑ Phe, Ile, Lys, Gly, Tyr, Ala, allo Thr, ↓ Cystine, threonic acid<br>Fatty acids: ↑ Stearic acid, glyceric acid, palmitic acid, linoleic acid, 2-monostearoyl-glycerol, ↓ Glycerol palmitate, hexadecanoic acid derivative<br>Carbohydrates: ↑ Lactic acid, di-TMS ethylene glycol, citric acid, myo-inositol glycerol-3-phosphate, 3-hydroxy butanoic acid, 2-hydroxy butanoic acid, ↓ Fructose, D-ribose 5-phosphate, glycerol, arabinose, malonic acid	↓ nonane, unknown (11), alkane (1)
Y. Zhang, 2016 [26]	Fatty acids: ↓ C16:1 (palmitoleic acid), C18:3 (linolenic acid), C18:2 (linoleic acid), C18:1 (oleic acid), C20:4 (arachidonic acid), C22:6 (docosahexanoic acid)	
H. Gu, 2015 [27]	Amino acids/ peptides: ↑ Asp, Glu, ↓ Gln /Lys, His, Arg, Ser, Tyr	
Zhu, 2014 [28]	Amino acids/ peptides: ↑ Hyp/ aminolevulinate, hippuric acid, leucic acid, ↓ His, 2-aminoadipate, N-acetylglycine, Met<br>Fatty acids: ↑ Glycocholate, glycochenodeoxycholate, ↓ Linolenic acid<br>Carbohydrates: ↑ Glyceraldehyde, maleic acid, ↓ Malonic acid/ 3-hydroxybutyrate (3HBA)	
F. Li, 2013 [29]	Fatty acids: ↑ LPC(16:0), ↓ Palmitic amide, oleamide, hexadecanedioic acid, octadecanoic acid, eicosatrienoic acid, myristic acid, LPA(16:0), LPA(18:0)	
Ritchie, 2013 [30]	Fatty acids: ↓ Gastrointestinal tract acid 446 (GTA-446)	
Tan, 2013 [31]	Amino acids/ peptides: ↓ 4-Hyp, Orn, Trp, Phe, Glu, urea<br>Fatty acids: ↑ Oleic acid<br>Carbohydrates: ↑ 2-hydroxybutanoic acid, 3-hydroxybutanoic acid, pyruvate	
Ikeda, 2012 [32]	Amino acids/ peptides: ↑ L-Ala, L-Gln, → L-Pro, L-Met, L-Glu, Asn<br>Fatty acids: ↑ Glycolic acid, thiodiglycolic acid, 3-hydroxy-propionic acid<br>Carbohydrates: ↑ Glucuronoic lactone, lactic acid, 3-hydroxy-isobutyric acid	
Leichtle, 2012 [33]	Amino acids/ peptides: ↓ Gly, Lys, Ala, Asp, His, Leu/Ile, Met, sarcosine, Thr, Tyr, Val	
Ma, 2012 [34]	Amino acids/ peptides: ↓ L-Val, L-Thr, Gly<br>Carbohydrates: ↑ 3-hydroxybutyric acid, ↓ ribitol ↓, 1-deoxyglucose	
Nishiumi, 2012 [35]	Amino acids/ peptides: ↑ Asp, kynurenine, cystamine<br>Carbohydrates: ↑ 2-hydroxybutyrate	

Ritchie, 2010 [36]	Fatty acids: ↓ C28H46O4 (446), C28H48O4 (448), C28H50O4 (450)
Ludwig, 2009 [37]	Fatty acids: → Acetate;<br>Carbohydrates: → Lactate, pyruvate, 3-hydroxybutyrate, acetoacetate
Plasma	
Liu, 2018 [38]	Amino acids/ peptides: ↑ Homocysteine
Nishiumi, 2017 [39]	Amino acids/ peptides: ↑ Orn, ↓ Trp, Lys<br>Fatty acids: ↑ Glycolic acid, ↓ Palmitoleic acid, 3-hydroxyisovaleric acid<br>Carbohydrates: ↑ Pyruvic acid, fumaric acid
Shen, 2017 [40]	Fatty acids: ↑ PG (34:0), SM (42:2), Cer (44:5), ↓ LPC (18:3), LPC (18:2), PE (O-36:3), PE (O-38:3), SM (38:8)
Crotti, 2016 [41]	Fatty acids: ↑ Decanoic acid
Cavia-Saiz, 2014 [42]	Amino acids/ peptides: ↑ L-Kynurenine
S. Li, 2013 [43]	Fatty acids: ↓ Saturated LPC, 18:2 LPC, SPC
Miyagi, 2011 [44]	Amino acids/ peptides: ↑ Ser, Ala, Ile, Leu, Lys, ↓ Val, Tyr, His, Trp, Arg
Okamoto, 2009 [45]	Amino acids/ peptides: ↓ Thr, Val, ↑ Glu, alpha-aminobutyric acid, Gln, Pro
Zhao, 2007 [46]	Fatty acids: 18:2-LPC ,18:1 LPC, unsat. LPC, sat. LPC
Urine	
Nakajima, 2018 [47]	Amino acids/ peptides: ↑ N1,N12-Diacetylspermine
Deng, Chang, 2017 [48]	Fatty acids: ↓ Carnitine<br>Carbohydrates: ↓ Succinic acid, ascorbic acid
Deng, Fang, 2017 [19]	Amino acids/ peptides: ↓ Ser, → β-Ala, p-Methylhistidine, Asn, trigonelline, His, Tyr<br>Fatty acids: → Butyrate, adipate<br>Carbohydrates: → Methanol, ethanol, 3-hydroxymandelate, benzoate, acetone, 3-Hydroxybutyrate, 3-Hydroxy-phenylacetate, 2-oxoglutarate
Wang, 2017 [49]	Amino acids/ peptides: ↓ Asn, Ala, Gln<br>Carbohydrates: ↑ Acetoacetate
Rozalski, 2015 [50]	Carbohydrates: ↑ 8-oxo-7,8-dihydroguanine, 8-oxo-7,8-dihydro-2'-deoxyguanosine, ↓ 5-hydroxymethyluracil
Wang, 2014 [51]	Amino acids/ peptides: ↓ Ser, → β-Ala, p-Methylhistidine, Asn, trigonelline, His, Tyr<br>Fatty acids: → Butyrate, adipate<br>Carbohydrates: →Methanol, ethanol, 3-hydroxymandelate, benzoate, acetone, 3-Hydroxybutyrate, 3-Hydroxy-phenylacetate, 2-oxoglutarate
Eisner, 2013 [16]	Amino acids/ peptides: ↑ Trigonelline, Tyr<br>Carbohydrates: ↓ Methanol, ↓ acetone
Hsu, 2013 [52]	Carbohydrates: ↑ Adenosine, cytidine, 3-methylcytidine, 1-methyladenosine, inosine, 2-deoxyguanosine
Yue, 2013 [17]	Fatty acids: ↓ C9H18N2O, C14H14N12O, C13H13NO4, C15H20NO5, O-octanoyl-R-carnitine, C19H35NO5, sphinganine, dihydrosphingosine, Unknown (1) 16H22O4
Chen, 2012 [53]	Amino acids/ peptides: ↑ Arg, Ile, Leu, Val, ↓ His, Met, Ser, Asp<br>Carbohydrates: ↑ Lactic acid, ↓ Citric acid, malic acid, succinate
Cheng, 2012 [54]	Amino acids/ peptides: ↑ Putrescine, 2-aminobutyrate, ↓ Kynurenate, hippurate<br>Fatty acids: ↑ Myristate

	Carbohydrates: ↓ Citrate, p-cresol	
Wang, 2010 [21]	Amino acids/ peptides: → Phenylacetylglutamine, leucylproline, aspartyllysine, prolylleucine<br>Fatty acids: → Acylcarnitine (C13:0), acetylcarnitine, acylcarnitine (C9:0), acylcarnitine (C9-OH), C11H20O4N2	Unknown (3)
Wang, 2010 [21]	Carbohydrates: ↑ Pseudouridine, cytidine, 2,2-dimethylguanosine, uridine, (1 unknown), 1-methyladenosine, N6-methyladenosine	
Johnson, 2006 [20]	Fatty acids: ↑ PGE-M	
Feng, 2005 [55]	Carbohydrates: ↑ Pseudouridine, 1-methylguanosine	
Hiramatsu, 2005 [56]	Amino acids/ peptides: ↑ N1,N12-Diacetylspermine	
Zheng, 2005 [57]	Carbohydrates: ↑ Pseudouridine, cytidine, 1-methyladenosine, inosine, 5-methyluridine, 1-methylinosine, 1-methylguanosine, N4-acetylcytidine, N2-methylguanosine, adenosine, N2,N2-methylguanosine, N6-methyladenosine, ↓ uridine, guanosine	
Feces		
Lin, 2016 [58]	Amino acids/ peptides: ↓ Gln, ↑ Pro, Ile/Leu/Val, Ala, Glu, dimethylglycine<br>Fatty acids: ↓ Acetate, propionate, butyrate<br>Carbohydrates: ↑ Succinate, lactate, ↓ Glucose	
Amiot, 2015 [59]	Amino acids/ peptides: ↓ Gln, Glu<br>Fatty acids: ↑ Valerate, propionate, butyrate, acetate<br>Carbohydrates: ↓ β-glucose	
Phua, 2014 [15]	Fatty acids: ↓ Linoleic acid<br>Carbohydrates: ↓ Nicotinic acid, fructose	
Bezabeh, 2009 [60]	Amino acids/ peptides: → Glu, Val, Ile<br>Fatty acids: → n-butyric acid, lipid	

Abbreviations: Ala, alanine; Arg, arginine; Asn, asparagine; Asp, aspartic acid (aspartate); Cer, ceramide; Cys, cysteine, Gln, glutamine; Glu, glutamic acid (glutamate); GTA-446, Gastrointestinal tract acid 446; His, histidine; Hyp, hydroxyproline; Ile, isoleucine; Leu, leucine; LPA, lysophosphatidic acid; LPC, lysophosphatidylcholine; Lys, lysine; Met, methionine; Orn, ornithine; PC, phosphatidylcholine, PE, phosphatidylethanolamine; PG, phosphatidylglycerol; PGE-M, Prostaglandin E2 metabolite; Phe, phenylalanine; Pro, proline; sat, saturated; Ser, serine; SM, sphingomyelin; SPC, sphingosylphosphorylcholine; Thr, threonine; Trp, tryptophan; Tyr, tyrosine; unsat, unsaturated; Val, valine.

Table S4. QUADAS tool.

First author, Year	Risk of bias				Concerns regarding applicability		
	Patient selection	Index test	Reference standard	Flow and timing	Patient selection	Index test	Reference standard
Dried blood spot							
Jing, 2017 [18]	☹	☺	☺	☺	☹	☺	☺
Serum							
Zhang, 2018 [22]	☹	☹	☹	? 😐	☹	☺	? 😐
Guo, 2017 [24]	☹	☹	? 😐	? 😐	☹	☺	? 😐
Hata, 2017 [25]	☹	☹	? 😐	? 😐	☹	☺	☺
Uchiyama, 2017 [23]	☹	☹	? 😐	? 😐	☹	☺	? 😐
Farshidfar, 2016 [14]	☹	☺	☺	☺	☹	☺	☺
Y. Zhang, 2016 [26]	☹	☺	? 😐	? 😐	☹	☺	? 😐
H. Gu, 2015 [27]	☺	☺	☺	☺	☺	☺	☺
Zhu, 2014 [28]	? 😐	☺	☺	☺	? 😐	☺	☺
F. Li, 2013 [29]	☹	☹	? 😐	? 😐	☹	☺	? 😐
Ritchie, 2013 [30]	? 😐	? 😐	☺	? 😐	☹	☺	☺
Tan, 2013 [31]	☹	☺	? 😐	? 😐	☹	☺	? 😐
Ikeda, 2012 [32]	☹	☹	☺	☺	☹	☺	☺
Leichtle, 2012 [33]	☹	☺	? 😐	? 😐	☹	☺	? 😐
Ma, 2012 [34]	☹	☹	? 😐	? 😐	☹	☺	? 😐
Nishiumi, 2012 [35]	☹	☺	? 😐	☺	☹	☺	☺
Ritchie, 2010 [36]	☹	☺	☺	? 😐	☹	☺	☺
Ludwig, 2009 [37]	☹	☹	? 😐	? 😐	☹	☺	? 😐
Plasma							
Liu, 2018 [38]	☹	☹	? 😐	? 😐	☹	☺	? 😐
Nishiumi, 2017 [39]	☹	☹	☺	? 😐	☹	☺	☺
Shen, 2017 [40]	☹	☹	? 😐	? 😐	☹	☺	? 😐
Crotti, 2016 [41]	☹	☹	☺	☺	☹	☺	☺
Cavia-Saiz, 2014 [42]	☹	☹	☺	☺	☹	☺	☺
S. Li, 2013 [43]	☹	☺	☺	☺	☹	☺	☺
Miyagi, 2011 [44]	☹	☺	? 😐	? 😐	☹	☺	? 😐
Okamoto, 2009 [45]	☹	☺	? 😐	☺	☹	☺	? 😐
Zhao, 2007 [46]	☹	☺	? 😐	? 😐	☹	☺	? 😐
Urine							
Nakajima, 2018 [47]	☹	☺	? 😐	? 😐	☹	☺	? 😐
Deng, Chang, 2017 [48]	☺	☺	☺	☺	☺	☺	☺
Deng, Fang, 2017 [19]	☺	☹	☺	☺	☺	☺	☺
Wang, 2017 [49]	☹	☺	? 😐	? 😐	☹	☺	? 😐
Rozalski, 2015 [50]	☹	☹	☺	☺	☹	☺	☺
Wang, 2014 [51]	☺	☺	☺	☺	☺	☺	☺
Eisner, 2013 [16]	☺	☺	☺	☺	☺	☺	☺
Hsu, 2013 [52]	☹	☹	? 😐	? 😐	☹	☺	? 😐
Yue, 2013 [17]	☹	☺	? 😐	? 😐	☹	☺	? 😐
Chen, 2012 [53]	☹	☹	? 😐	☺	☹	☺	☺
Cheng, 2012 [54]	☹	☺	? 😐	? 😐	☹	☺	? 😐
Wang, 2010 [21]	☹	☺	? 😐	? 😐	☹	☺	? 😐
Johnson, 2006 [20]	☹	☹	☺	☺	☹	☺	☺
Feng, 2005 [55]	☹	☹	? 😐	? 😐	☹	☺	? 😐
Hiramatsu, 2005 [56]	☹	☹	? 😐	? 😐	☹	☺	? 😐
Zheng, 2005 [57]	☹	? 😐	☺	☺	☹	☺	☺
Feces							
Lin, 2016 [58]	☹	☺	☺	? 😐	☹	☺	☺
Amiot, 2015 [59]	☺	☺	☺	☺	☺	☺	☺
Phua, 2014 [15]	☹	☺	? 😐	☺	☹	☺	☺
Bezabeh, 2009 [60]	? 😐	☺	☺	☺	? 😐	☺	☺

Note: ☹ high risk; ☺ low risk; **?** 😐 unclear risk.

© 2018 by the authors. Licensee MDPI, Basel, Switzerland. This article is an open access article distributed under the terms and conditions of the Creative Commons Attribution (CC BY) license (http://creativecommons.org/licenses/by/4.0/).
